# NF-kappaB activation: Tax sumoylation is out, but what about Tax ubiquitination?

**DOI:** 10.1186/1742-4690-9-78

**Published:** 2012-09-25

**Authors:** Gutian Xiao

**Affiliations:** 1 University of Pittsburgh Cancer Institute, University of Pittsburgh Medical Centers, Pittsburgh, PA 15213, USA; 2 Department of Microbiology and Molecular Genetics, University of Pittsburgh School of Medicine, Pittsburgh, PA 15261, USA

**Keywords:** ATL, IKK, HTLV, Leukemia, NF-κB, Nuclear body, Retrovirus, SUMO, Tax, Taxisome, Tumroigenesis, Ubiquitin

## Abstract

Activation of the NF-κB transcription factors by the viral protein Tax plays a vital role in the pathogenesis of diseases associated with human T-cell lymphotropic virus type I (HTLV-I). The Tax oncoprotein undergoes constitutive K63-linked ubiquitination and sumoylation; however, the roles and molecular mechanisms of these post-translational modifications in Tax-mediated NF-κB activation are being debated. Here, we discuss our current understanding of Tax activation of NF-κB, with a focus on the controversies and the challenges that we are facing.

## Background

Human T-cell lymphotropic virus type I (HTLV-I) is the etiological agent of adult T-cell leukemia/lymphoma (ATL) and a number of inflammatory diseases. The pathogenesis of these HTLV-I-associated diseases, particularly the leukemogenesis of ATL, is mainly mediated by the viral regulatory protein Tax (reviewed in [1]). Molecular, genetic and pharmacologic studies have demonstrated that Tax exerts its pathogenic efforts largely through persistent activation of NF-κB transcription factors [2-4].

**Figure 1:**
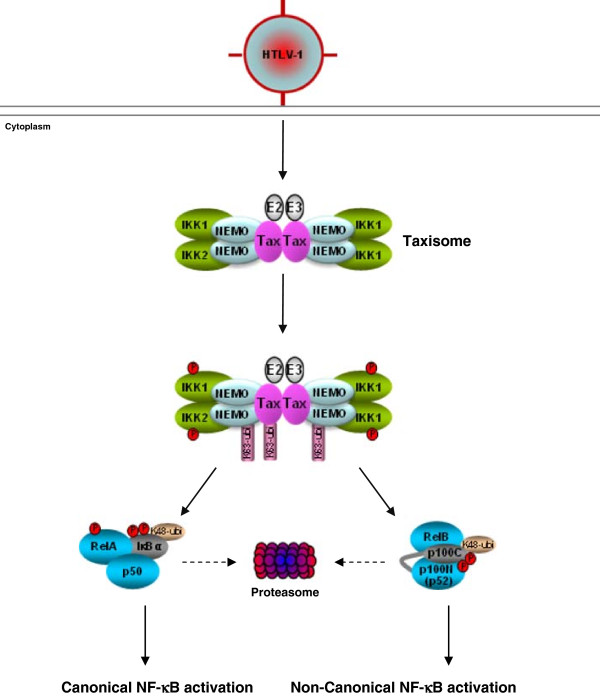
**A model depicting the mechanisms of Tax-mediated IKK/NF-κB activation.** 1. Tax dimerizes with each other and binds to NEMO to recruit two IKK complexes and also an E2 ubiquitin conjugating enzyme and an E3 ubiquitin ligase to form the Taxisome; 2. Some Tax and NEMO proteins within the Taxisome are polyubiquitinated *via* ubiquitin lysine 63 (K63) by the E2 ubiquitin conjugating enzyme and E3 ubiquitin ligase; 3. K63-linked ubiquitination of Tax and/or NEMO leads to catalytic activation of IKK1 and IKK2 within the Taxisome *via* two alternative mechanisms: K63-linked polyubiquitin chains either act as the platform to recruit IKKK to phosphorylate the activation loops of IKK1 and IKK2, or interact with each other and the components within the Taxisome to cause structural alterations of the Taxisome and trans-autophosphorylation of IKK1 and IKK2; 4a. Activated IKK2 and IKK1 respectively phosphorylate IκBs and RelA, leading to canonical NF-κB activation; 4b. Activated IKK1 is selectively recruited by Tax (through NEMO) into the p100 complex to phosphorylate p100, resulting in non-canonical NF-κB activation (see text for details and other possible or alternative mechanisms involved in the activation of IKK/NF-κB by Tax).

Like most NF-κB inducers, Tax activates NF-κB through the IκB kinase (IKK) complex, which contains two catalytic components, IKK1 (also known as IKKα) and IKK2 (IKKβ), and a regulatory component, NEMO (IKKγ) (Figure 1). Tax physically interacts with NEMO, *via* the leucine-repeat motif of Tax and two homologous leucine zipper domains within NEMO, resulting in persistent IKK and NF-κB activation [5,6]. Except for its cytoplasmic role in IKK activation, Tax has been reported to recruit RelA (also known as p65, the prototypic member of NF-κB) as well as other cellular factors into interchromatin granules to form discrete transcriptional hot spots termed ‘Tax nuclear bodies’ for full NF-κB transcriptional activation [7,8]. Based on these trailblazing studies, much effort has been devoted to pinpoint the detailed mechanisms by which Tax activates IKK and NF-κB in the past eight years. In particular, it has been proposed that K63-linked polyubiquitination of Tax is critical for Tax-binding to NEMO and subsequent IKK activation and RelA nuclear translocation, while Tax sumoylation is required for the formation of Tax nuclear bodies and RelA-dependent transcription [9] and references therein]. It has been also proposed that Tax ubiquitination and sumoylation collaboratively facilitate Tax nucleocytoplasmic shuttling and thereby contribute to Tax-mediated NF-κB activation [9] and references therein]. However, these proposals have faced major challenges ever since (or even before) they were raised.

### New data and new challenge

Using a Tax point mutant, Bonnet *et al*. recently reported that Tax sumoylation and nuclear body formation are dispensable for Tax-mediated IKK/NF-κB activation [9]. In search of functional motifs in Tax, they mutated the potential TRAF (TNF receptor associate factor)-binding sequence PxQxT within Tax, because TRAF proteins are well-known for their involvement in the induction of IKK/NF-κB activity. Interestingly, mutation of this motif has no effect on Tax binding to TRAF proteins, but significantly diminishes the sumoylation of Tax and greatly decreases the formation of Tax nuclear bodies. Remarkably, the mutant retains other abilities of Tax, such as cytoplasmic and nuclear distributions, K63-linked ubiquitination, NEMO binding, IKK catalytic activation, NF-κB transcriptional activation as well as CREB activation, another major function of Tax that is involved in viral gene expression. These findings clearly suggest that both Tax sumoylation and nuclear body formation are not key determinants for Tax-mediated NF-κB activation. However, these studies cannot rule out the possibility that Tax sumoylation and/or nuclear bodies may be involved in the transcriptional regulation of some specific NF-κB target genes.

### Other debated issues and discussions

In contrast to its sumoylation, Tax ubiquitination is likely involved in Tax-mediated NF-κB activation. However, many fundamental issues on Tax ubiquitination still remain unclear or controversial. For example, the mechanisms of how Tax is ubiquitinated are being debated. In line with their role as the E2 ubiquitin conjugating enzyme and E3 ubiquitin ligase in promoting K63-linked ubiquitination of some molecules important in IKK and NF-κB activation, Ubc13 and TRAF2, 5 or 6 have been suggested to be responsible for the K63-linked ubiquitination of Tax [10,11]. However, opposite findings have also been reported showing that Ubc13 and, at least, TRAF6 are not required for Tax ubiquitination [12] and references therein]. Similarly, whether and which deubiquitinase(s) are involved in the negative regulation of Tax ubiquitination are also being debated. Two deubiquitinases CYLD and USP20 have been reported to remove the K63-linked polyubiquitin chains from Tax [13,14]. Intriguingly, those reports showed a strong interaction of the deubiquitinases with the unmodified Tax, but not with ubiquitinated Tax. Even more confusing are studies from different research groups reporting that, at least, CYLD is not involved in Tax deubiquitination [12] and references therein].

Perhaps, the most important and controversial issue in the field is the molecular mechanism by which the ubiquitin modification contributes to Tax activation of NF-κB, although the evidence directly supporting the importance of Tax ubiquitination in Tax activation of IKK and NF-κB is still lacking. Three different mechanisms have been proposed, but all of them are being debated. The first one is that Tax ubiquitination is critical for Tax binding to NEMO and therefore important for IKK and NF-κB activation. This conclusion was based on the data that ubiquitination-defective mutant of Tax loses the ability in NEMO binding and NF-κB induction, and fusion of ubiquitin to the mutant partially rescues NF-κB activation in gene reporter assays [9] and references therein]. The data are interesting and similar to those previously used to reach the conclusion that Tax sumoylation is important for Tax-mediated NF-κB activation. However, the data, also like those for Tax sumoylation, are too preliminary to draw a firm conclusion. The defects of the Tax mutant in NEMO binding and NF-κB induction are not necessarily linked to its inability in ubiquitination, because Tax is highly mutation sensitive. Similarly, the gain-of-function by ubiquitin fusion may or may not support the role of Tax ubiquitination in Tax-mediated IKK/NF-κB activation, as ubiquitin chain itself is able to activate IKK and NF-κB [15]. In addition, these studies failed to show the interaction between ubiquitinated Tax and NEMO. Nevertheless, the fact that the unmodified form of Tax physically interacts with NEMO under all the conditions examined (either *in vitro* or *in vivo*; HTLV-I related or unrelated; in the presence or absence of the ubiquitination system) strongly argues that Tax ubiquitination is dispensable for Tax binding to NEMO. The second proposed mechanism is that Tax ubiquitination is required for Tax to recruit the IKK complex (through NEMO) into particular perinuclear compartment for IKK activation. Some studies have suggested the particular subcellular locations are centrosomes, while others implied endoplasmic reticulum (ER) or Golgi-associated structures [9] and references therein]. Regardless of the differences, the conclusions were largely based on the co-localizations of Tax or Tax mutants with IKK at these sites by microscope analysis and subcellular fractionation assays, but not based on the functional studies that can directly support the hypotheses. Moreover, a recent study showed that purified recombinant Tax activates IKK that exists in the cytosolic extracts of cells [12]. These data strongly argue that intact cellular structures, including centrosomes, ER and Golgi, are not required for Tax-mediated IKK activation. The third proposed mechanism is that Tax ubiquitination is required for Tax to recruit IKK kinase (IKKK) to phosphorylate IKK for its catalytic activation. Several mitogen-activated protein kinase kinase kinases (MAP3Ks), such as MEKK1, NIK, Tpl2 and TAK1, have been linked to Tax-mediated IKK/NF-κB activation. These MAP3Ks are well-known IKK activators. But again, opposite findings have also been reported showing that none of these kinases are required for Tax-mediated IKK/NF-κB activation, although some of them are indispensible for Tax-mediated activation of signaling pathways other than IKK/NF-κB [12] and references therein].

## Conclusions

Despite extensive studies over the past two and half decades, our understanding of the mechanisms of Tax-mediated NF-κB activation is still far from complete. As discussed above, many conflicting results have been reported and accordingly many conflicting conclusions have been drawn. Regardless of the conflicts, a plausible model of Tax-mediated NF-κB activation could be formulated (Figure1). By self-dimerization and selective association with NEMO *via* specific binding motifs within Tax and NEMO, Tax brings different IKK complexes and possibly also other proteins (such as E2 ubiquitin conjugating enzyme and E3 ubiquitin ligase) together to form the Tax/IKK signalsome/signaling complex (Taxisome) with a gel filtration size larger than 600 kDa. Some Tax and NEMO proteins within the signalsome are polyubiquitinated by the E2 ubiquitin conjugating enzyme and E3 ubiquitin ligase. The anchored K63-linked polyubiquitin chains then act as the platforms for recruiting IKKK to phosphorylate the activation loops of IKK1 and IKK2, resulting in the catalytic activation of IKK. Alternatively, the polyubiquitin chains interact with each other and/or the components within the Taxisome, leading to the structural alterations of the Taxisome and the trans-autophosphorylation and activation of IKK1 and IKK2. The activated IKK in turn phosphorylates IκBs (by IKK2) and also RelA (by IKK1), resulting in β-TrCP-mediated K48-linked ubiquitination and proteasomal degradation of IκBs, nuclear translocation of NF-κB dimers including those containing the phosphorylated RelA, and ultimately transcriptional activation of NF-κB target genes (mechanism leading to canonical NF-κB activation). In parallel, Tax specifically recruits the activated IKK1 (through NEMO) into the p100 complex to phosphorylate p100, leading to p100 K48-linked ubiquitination by the β-TrCP ubiquitin ligase and proteaomal processing to generate NF-κB2 p52. The newly generated p52 together with its NF-κB binding partners translocates into the nucleus, where they induce or repress gene expression (mechanism leading to non-canonical NF-κB activation). Although it seems that intact cellular structures, including centrosomes, ER and Golgi, are not required for Taxisome formation and subsequent IKK activation, these structures or some of them may facilitate these important events. Similarly, Tax sumoylation and nuclear body formation may also contribute to Tax induction or repression of some specific NF-κB target genes, although they are dispensable for overall NF-κB activation. It is also noteworthy that Tax ubiquitination may be not involved in the IKK and NF-κB activation. The ubiquitination of Tax may be just the byproduct of ubiquitination of other components within the Taxisome such as NEMO, and *vice versa*.

## Abbreviations

ATL: Adult T-cell leukemia/lymphoma; CREB: CAMP response element-binding protein; β-TrCP: β-transducing repeat-containing protein; CYLD: Cylindromatosis protein; ER: Endoplasmic reticulum; HTLV-I: Human T-cell lymphotropic virus type I; IκBs: Inhibitors of IκB; IKK: IκB kinase; IKKK: IKK kinase; MAP3K: Mitogen-activated protein kinase kinase kinase; MEKK1: Mitogen-activated protein/extracellular signal-regulated kinase (MEK) kinase 1; NEMO: NF-κB essential modulator; NF-κB: Nuclear factor-κB; NIK: NF-κB inducing kinase; TAK1: Transforming growth factor-β (TGFβ)-activated kinase 1; Taxisome: Tax/IKK signalsome/signaling complex; Tpl2: Tumor progression locus 2; TRAF: Tumor necrosis factor (TNF) receptor associate factor; Ubc13: Ubiquitin-conjugating enzyme 13; USP20: Ubiquitin-specific peptidase 20.

## Competing interests

The author declares no competing financial interests.

## Authors’ contributions

GX conceived the topic, and wrote and approved the manuscript.
